# Commentary: Chinese as a Second Language Multilinguals' Speech Competence and Speech Performance: Cognitive, Affective, and Sociocultural Perspectives

**DOI:** 10.3389/fpsyg.2022.935909

**Published:** 2022-06-20

**Authors:** Chunyan Ma, Shuang Zhang, Chili Li

**Affiliations:** ^1^Department of Education, Faculty of Arts and Social Sciences, New Era University College, Kajang, Malaysia; ^2^School of Humanities, Wuhan University of Engineering Science, Wuhan, China; ^3^School of Foreign Languages, Hubei University of Technology, Wuhan, China

**Keywords:** Chinese as a second language, multilinguals' speech competence and speech performance, cognitive, affective, sociocultural perspectives

The recently-published book entitled *Chinese as a Second Language Multilinguals' Speech Competence and Speech Performance: Cognitive, Affective, and Sociocultural Perspectives* by Sun (2020) aroused our interest, for its comprehensive overview in crystallizing the disposition of Chinese as a second language (CSL) multilinguals' speech competence and performance. This book consists of eight chapters. Chapter 1 describes the background of the research. A conceptual framework is then established in Chapter 2 to explain the relevant terms. The two concepts regarding speech competence and speech performance are introduced in Chapter 3. Chapter 4 thoroughly reviews the literature on the research of L2 learning and speaking from the cognitive, affective, and sociocultural perspectives. After that, the design of the study is described in Chapter 5. In Chapter 6 and Chapter 7, the quantitative and qualitative results are reported. Then, Chapter 8 concludes the manuscript concerning the implications and limitations of the study as well as presenting suggestions for further research. This book deserves recommendation and comment because of its significant theoretical, practical, and methodological advantages.

This book focuses on CSL multilinguals' speech competence and speech performance, which is a timely contribution to the collected scholarship of research on CSL learners. In recent decades, Chinese has gradually developed as an influential language taught and learnt all over the world (Gong et al., 2018, 2020a; Sun, 2021). It has been reported that more than 70 countries have incorporated Chinese into their national education systems (Gong et al., 2020b; Li et al., 2021). In 2018, a total of 437,331 people took HSK (Hanyu Shuiping Kaoshi) and HSKK (Hanyu Shuiping Kouyu Kaoshi: Chinese Speaking Proficiency Test) exams worldwide, of which 30,407 took HSKK exams, accounting for 6.95 percent of the total. Compared with HSK, the number of examines in HSKK is still relatively small (Ding et al., 2021). Given the incremental CSL population around the globe, it is essential to explore how this group of learners learn the target language. Existing research on speech competence and performance mainly focuses on learners of English as a second language (Gao and Liu, 2015; Yu and Liu, 2020; Xu and Long, 2022). However, there is a paucity of effort made in this regard on CSL learners (Wen, 2018; Ding et al., 2021). Therefore, this book enriches the body of literature on CSL learners, their features of speech competence and speech performance in particular. Theoretically, the findings yielded from this mixed-methods study validated and lent empirical support to an array of theories, namely, Affective Filter Hypothesis (Krashen, 1982), L2 Willingness to Communicate (WTC) model (MacIntyre et al., 1998), L2 Motivational Self System (Dörnyei, 2009) and L2 speech production model (Segalowitz, 2010). Specifically, it is revealed in this book that the development and production of L2 Chinese speaking tends to be influenced by a body of affective factors, such as anxiety, speaking self-efficacy, motivation, and WTC. It is also indicated that L2 WTC is related to learners' speech competence and speech performance. Furthermore, it has been suggested that the consistency between ideal speech competence and actual speech performance is connected with the learners' Chinese language proficiency. Lastly, this study manifests that positive attitudes toward L2 classes, L2 communities, and L2 cultural interest are important to the improvement of CSL multilinguals' L2 Chinese speech competence and performance.

Practically, it is implied in this study that CSL learners and teachers should acknowledge the inevitability of the discrepant and unbalanced development of speech competence and performance in the process of L2 learning due to the influence of various cognitive, affective, and sociocultural factors. To minimize such a discrepancy, it is advisable for learners to enrich their repertoire of L2 Chinese speaking strategies. For example, learners are encouraged to grasp opportunities to interact with native speakers. When feeling anxious in speaking the target language in front of others, they are suggested to make full preparations such as self-rehearsal in advance. Moreover, the book has underscored the importance and necessity of paying attention to the preferences of different learning styles among the CSL learners. Thus, it is suggested that teachers should take into full consideration the learners' preferred styles when designing their teaching. Teachers could provide guidance, support, and encouragement for learners in the development of their speech competence and speech performance.

Methodologically, the study reported in this book combines the quantitative and qualitative techniques, which shall provide complementarity and corroboration for the findings. For instance, the quantitative results reveal that the discrepancy between speech competence and performance among the CSL multilinguals might result from the influence of cognitive, affective, and sociocultural factors. This finding is further corroborated in the qualitative results. Besides, this study applies standardized tests to measure the participants' speech competence and performance, instead of relying on the traditional practice of asking the respondents to self-report. Additionally, the author utilizes interviews and focus groups to triangulate the whole study, which could guarantee the reliability and validity of the results. Therefore, this study could methodologically offer paradigmatic reference for future research of such kind.

Based on the aforementioned advantages, this book is worthy of being commented on and shared to larger reader groups. Nevertheless, this book has certain disadvantages. Firstly, the theoretical frameworks applied in this study used to be mainly directed for English as a second language acquisition. Chinese, as a tonal language, differs from English in terms of its pronunciation and spelling system. The uniqueness of Chinese language thus warrants further validation of the results in other contexts. Secondly, a majority of the research participants including both the advanced and intermediate groups were CSL-related majors. Those CSL learners of other disciplines were not represented in the sampled population. Thirdly, this study reveals the potential influence of multilingualism upon the speech competence and performance of the CSL learners. However, the mechanism on how multilingualism exerts its impact from the cognitive, affective, and sociocultural perspectives is unknown. It is recommended that future studies can take these issues into consideration.

In conclusion, though this book has its shortcomings, these tiny limitations do not undermine its contributions. The book is very instructive, thought-provoking and illuminating with its theoretical, practical and methodological merits. With careful reading, readers shall definitely obtain a deeper understanding of the differences between CSL multilingual' speech competence and speech performance, and acquire a fuller scenario of the factors that might exert influence upon CSL multilinguals' speech competence and speech performance from cognitive, affective, and sociocultural perspectives. Therefore, this book is strongly recommended to those who are keen on exploring the acquisition of Chinese as a second/foreign language, the speech competence and performance among CSL multilinguals in particular, from a comprehensive approach.

## Author Contributions

CM drafted the General Commentary. SZ helped CM to select the commented book, provided insights and suggestions during her writing, and CL did the revision for the text. All authors contributed to the article and approved the submitted version.

## Conflict of Interest

The authors declare that the research was conducted in the absence of any commercial or financial relationships that could be construed as a potential conflict of interest.

## Publisher's Note

All claims expressed in this article are solely those of the authors and do not necessarily represent those of their affiliated organizations, or those of the publisher, the editors and the reviewers. Any product that may be evaluated in this article, or claim that may be made by its manufacturer, is not guaranteed or endorsed by the publisher.
